# Mutation in the *SLC2A9* Gene: A New Family with Familial Renal Hypouricemia Type 2

**DOI:** 10.1155/2021/4751099

**Published:** 2021-09-23

**Authors:** Christian Maalouli, Karin Dahan, Arnaud Devresse, Valentine Gillion

**Affiliations:** ^1^Division of Nephrology, Cliniques Universitaires Saint-Luc, Université Catholique de Louvain, Brussels, Belgium; ^2^Division of Human Genetics, Cliniques Universitaires Saint-Luc, Université Catholique de Louvain, Brussels, Belgium; ^3^Center of Human Genetics, Institut de Pathologie et de Génétique, Charleroi, Belgium

## Abstract

Familial renal hypouricemia is a rare genetic disorder characterized by a defect in renal tubular urate reabsorption. Some patients present with exercise-induced acute kidney injury and nephrolithiasis. Type II is caused by mutations in the *SLC2A9* gene. Here, we report the case of a young patient who developed acute kidney injury after exercise secondary to familial renal hypouricemia type II. The same mutation was found in other asymptomatic members of his family. We review the medical literature on this condition. This case highlights the importance of considering uric acid disorders in the work-up of acute kidney injury after exercise.

## 1. Introduction

Hypouricemia may be caused by decreased uric acid production or increased uric acid clearance [1–4]. The latter may be due decreased renal tubular reabsorption secondary to acquired disorders such as Fanconi syndrome or inherited disorders such as familial renal hypouricemia (RHUC) [1–6]. Unlike hyperuricemia and gout, hypouricemia has been thought as a disorder with no clinical significance. However, individuals with RHUC may present with exercise-induced acute kidney injury and nephrolithiasis [1–10]. Here, we report the case of a patient who developed acute kidney injury after strenuous exercise. The work-up has led to the discovery of a new family with RHUC type II.

## 2. Case Presentation

A 20-year-old man presented to the nephrology department with acute pain of both flanks which started after a physical exercise at school. His past medical history was unremarkable. He was not taking any medications or illicit drugs, and he denied smoking and consuming alcohol.

There was no abnormal finding in his physical examination. Vital signs were normal. Laboratory tests on admission showed acute kidney injury with a creatine kinase level of only twice the upper limit of the normal (Table 1). Urinalysis was bland, and the proteinuria was within the range (Table 1). Contrast-enhanced computed tomography showed decreased enhancement of the kidneys without any abnormality of the vessels and showed no urolithiasis. After conservative therapy including intravenous fluid administration, the renal function recovered gradually and returned to the normal at the fourth day after admission.

The patient reported that he was admitted 4 months ago in the emergency room of another hospital for a similar pain which occurred also after a strenuous exercise. Although laboratory tests showed acute kidney injury, he was discharged without any further investigation.

Laboratory tests were marked by severe hypouricemia. The elevated fractional excretion of urate (119%; *N* < 10%) suggested a highly increased urinary excretion of uric acid with tubular secretion. Blood and urinary tests showed no argument for Fanconi syndrome or syndrome of inappropriate antidiuretic hormone. Interesting enough, severe hypouricemia was also found in the laboratory tests of his mother and one of his maternal aunts. The occurrence of two episodes of acute kidney injury following intense exercise with the presence of hereditary severe hypouricemia highly suggested exercise-induced acute kidney injury due to familial renal hypouricemia.

**Figure 1:**
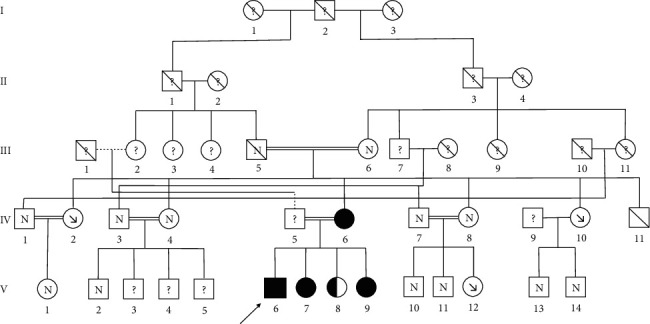
Pedigree of the patient. Solid symbols denote family members with homozygous mutation; half-solid denote heterozygous mutation. “N” denotes family members with normal uric acid level; “↘” denotes severe hypouricemia. “?” denotes family members with no laboratory data. Circles represent female family members, squares represent male family members, and crosses represent dead family members. Double lines indicate consanguineous marriage. Arrow indicates the index patient.

This index patient (V6) is a member of a highly consanguineous Pakistani family with cousin marriages over two successive generations (Figure 1). DNA sequencing by next-generation sequencing identified an homozygosity for a class 5 variant of the *SLC2A9* gene (NM_020041.3):c.646G > A, p.(Gly216Arg) confirmed by the Sanger technique [5]. Genetic testing of the patient's mother revealed the same homozygous mutation. Severe hypouricemia with the familial homozygous mutation was found in two of his clinically unaffected sisters (V7 and V9). The third sister (V8) is heterozygous for the mutation and is presenting with normal uric acid level. Profound hypouricemia was also measured in one of his aunts (IV2) and one of his cousins (V12). All of these analyses confirmed an autosomal recessive mode of inheritance. Besides the index patient, no medical history was known in all of the family members except his grandfather (III5) who suffered from kidney stones.

## 3. Discussion

RHUC is a rare autosomal recessive genetic disease caused by impaired renal tubular urate transport [1–10]. Less common than type 1 caused by mutations in the *SLC22A12* gene encoding the major uric acid transporter URAT1 (OMIM #220150), type 2 (RHUC2, OMIM #612076) is caused by mutations in the *SLC2A9* gene which encodes GLUT9 [1–5, 7–10]. Its long variant GLUT9L is the only major urate efflux transporter at the basolateral membrane, explaining why homozygous loss-of-function mutations of GLUT9 cause a total defect of uric acid tubular absorption [1, 4, 7–9]. To date, no more than 15 families have been described [1–4, 8, 9]. Although most patients are asymptomatic, the major complications are exercise-induced acute kidney injury (EIAKI) and urolithiasis [1–10]. EIAKI often follows intense exercise and is mostly described in males [4, 8, 10]. It may be secondary to excessive oxidative stress leading to renal vasoconstriction or precipitation of uric acid in the tubules [1, 3, 4, 8, 10]. When compared to type 1, patients with type 2 appear to have much higher renal excretion of uric acid and to be more vulnerable for EIAKI and nephrolithiasis [1, 9]. Measures to prevent these complications include limiting anaerobic physical exercise and maintaining adequate fluid intake [8–10]. Allopurinol may be proposed in specific cases [2–4, 9, 10].

## Figures and Tables

**Table 1:** Laboratory findings.

Test		Unit	Value at presentation	Normal range
*Serum*	CRP	mg/l	3.3	<5
	Creatinine	mg/dl	1.9	0.6–1.3
	Urea	mg/dl	60	17–48
	Uric acid	mg/dl	0.2	3.5–7.2
	Sodium	mmol/l	139	135–145
	Potassium	mmol/l	5.2	3.5–5.0
	Bicarbonate	mmol/l	22	23–29
	Calcium	mmol/l	2.3	2.2–2.5
	Phosphate	mmol/l	0.9	0.8–1.45
	Hemoglobin	g/dl	15.3	13–18
	WBC	/*μ*l	12310	3500–11000
	Neutrophils	/*μ*l	9630	1500–6700
	Platelets	/*μ*l	170000	150000–450000
	LDH	U/l	262	135–225
	CK	U/l	640	40–300

*Urine*	RBC	/*μ*l	2	<25
	WBC	/*μ*l	20	<25
	Creatinine	g/24h	0.98	0.8–2.5
	Protein	g/24h	0.08	<0.15
	Sodium	mmol/24h	216	40–220
	Potassium	mmol/24h	34	25–125
	Uric acid	mg/24h	234	250–750

Abbreviations: CRP, C-reactive protein; WBC, white blood cell; RBC, red blood cell; LDH, lactate dehydrogenase; CK, creatine kinase.

## Data Availability

The datasets used during the current study are available from the corresponding author on reasonable request.
